# Human Platelet Lysates‐Based Hydrogels: A Novel Personalized 3D Platform for Spheroid Invasion Assessment

**DOI:** 10.1002/advs.201902398

**Published:** 2020-02-11

**Authors:** Cátia F. Monteiro, Sara C. Santos, Catarina A. Custódio, João F. Mano

**Affiliations:** ^1^ Department of Chemistry CICECO University of Aveiro Campus Universitário de Santiago 3810‐193 Aveiro Portugal

**Keywords:** humanized 3D invasion models, hydrogels, in vitro disease models, multicellular tissue spheroids, platelet lysates

## Abstract

Fundamental physiologic and pathologic phenomena such as wound healing and cancer metastasis are typically associated with the migration of cells through adjacent extracellular matrix. In recent years, advances in biomimetic materials have supported the progress in 3D cell culture and provided biomedical tools for the development of models to study spheroid invasiveness. Despite this, the exceptional biochemical and biomechanical properties of human‐derived materials are poorly explored. Human methacryloyl platelet lysates (PLMA)‐based hydrogels are herein proposed as reliable 3D platforms to sustain in vivo‐like cell invasion mechanisms. A systematic analysis of spheroid viability, size, and invasiveness is performed in three biomimetic materials: PLMA hydrogels at three different concentrations, poly(ethylene glycol) diacrylate, and Matrigel. Results demonstrate that PLMA hydrogels perfectly support the recapitulation of the tumor invasion behavior of cancer cell lines (MG‐63, SaOS‐2, and A549) and human bone‐marrow mesenchymal stem cell spheroids. The distinct invasiveness ability of each cell type is reflected in the PLMA hydrogels and, furthermore, different mechanical properties produce an altered invasive behavior. The herein presented human PLMA‐based hydrogels could represent an opportunity to develop accurate cell invasiveness models and open up new possibilities for humanized and personalized high‐throughput screening and validation of anticancer drugs.

## Graphical abstract

Human methacryloyl platelet lysates (PLMA)‐based hydrogels have been recently proposed as a new cost‐effective and biologically relevant 3D platform. PLMA hydrogels are demonstrated to support invasion mechanisms of different human cell spheroids allowing a distinct invasiveness ability depending on their mechanical properties, thus validating their potential to develop accurate cell invasiveness models.

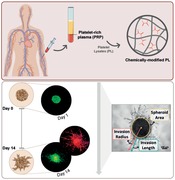

## 1 Introduction

The complexity and dynamism of the native tissue microenvironment are actively involved into distinct physiological and pathological events, where cell–cell and cell–extracellular matrix (ECM) communication plays a crucial role.^1^, ^2^ In cancer, tumor cell signaling triggers the deregulation of the spatiotemporal ECM remodeling that maintain the tissues integrity, favoring tumor cell migration and invasion into neighborhood regions and supporting metastasis development.^3^, ^4^ Effectively, a large number of cancer‐related deaths are derived from these cellular processes and therefore understanding tumor invasion mechanisms is a focus in cancer research.

Conventional 2D in vitro cultures have been the most widely used platforms for anticancer drug screening and validation. Despite their simplicity and quickness, they have recognized limitations in recapitulating in vivo behaviors which is reflected in misleading preclinical results.^5^, ^6^ 3D cell culture systems have been explored in cancer research field as a more complex and predictable in vitro model able to mimic the human in vivo cellular behavior in a more realistic way.^6^, ^7^, ^8^, ^9^ The biochemical, physical, and mechanical cues offered by in vitro 3D models maintain cell morphology and polarity while preserving cell surface receptors expression (e.g., integrins) involved in signaling pathways such as tumor metastatic cascade and therapy resistance.^10^, ^11^, ^12^, ^13^ The potential to provide fundamental insights on biomedical research has stimulated the interest of applying 3D cultures in preclinical assays, thus encouraging technological advances to achieve the implementation of high‐throughput and high‐content screening techniques.^14^, ^15^, ^16^, ^17^, ^18^

Multicellular spheroids are the most relevant 3D platform, largely contributing to the current knowledge of the tissues pathophysiology. Concerning tumor spheroids, the in vivo‐like cellular arrangement stimulates a set of biological conditions similar to those found in a native tumor mass. Oxygen, nutrients, and pH gradients are some of the spheroid‐reproduced chemical aspects that resemble the in vivo phenotypic heterogeneity of an avascular tumor.^19^, ^20^ These heterogeneous tumor cell populations are characterized by cellular proliferation and metabolic activity gradients modulated by distinct protein and gene expression profiles related with the invasiveness ability of tumor cells.^19^, ^21^ Contrary to 2D cultures, the native cell polarity is mimicked in these classic 3D models and preserved during the cell invasion process.^21^, ^22^ Boyden chamber is the most widely used in vitro standard assay to assess tumor invasion.^23^, ^24^, ^25^, ^26^ However, spheroids embedded into an ECM hydrogel have been proposed as a highly reproducible and automated system to develop accurate in vitro invasion models.^18^, ^27^

In vivo cell invasiveness ability is dependent on cell type and its environment and, with regard to the 3D in vitro invasion models, the method of spheroid generation and the physicochemical cues of the surrounding matrix have a huge effect on cell invasive behavior.^28^, ^29^ Numerous biomimetic materials, from natural origin to synthetic polymers, have been used to recreate the ECM in 3D in vitro models.^30^, ^31^ Typically, natural‐based scaffolds are biochemically similar to the native tissue to be reproduced/replaced.^32^ Synthetic ones have easily controllable properties; however, chemical functionalization is critical to improve their biochemical cues. Therefore, the search for new biomimetic materials that accurately mimic the natural ECM and, at the same time, provide reproducible cell invasive behavior is essential to an in‐depth comprehension of invasive mechanisms and future application as platforms for drug screening. Reconstituted basement membrane (rBM) of murine Engelbreth‐Holm‐Swarm sarcoma and type I collagen are the most used natural biomaterials so far. However, they exhibit noteworthy drawbacks regarding its reproducibility, difficult handling, and the animal origin that can induce an immune response and limit their clinical application.^30^, ^32^ Our research group recently proposed a novel 3D in vitro platform made of photo‐crosslinkable human platelet‐rich plasma (PRP)‐derived hydrogels for the development of complex and reliable tissue models.^33^ This humanized biomaterial follows the animal‐free tendency, being rich in allogenic fibrous and adhesive proteins, growth factors, and other bioactive factors that provide specific cues of human tissues microenvironment.^34^ Besides their remarkable biochemical properties for tissue engineering applications, methacryloyl platelet lysates (PLMA)‐based hydrogels demonstrated to be a simple and cost‐effective platform with highly tunable mechanical properties, supporting human‐derived cell growth and invasion.^33^ The combination of human‐specific biochemical and mechanical cues provides a physiologically relevant tissue‐like environment to develop tissue models mimicking cell‐intrinsic behavior, which can improve the predictivity of pharmacokinetic responses.^35^, ^36^ PLMA‐based hydrogels have the potential to increase the predictive value of drug screening and validation models over the existing animal‐derived platforms, which can include biochemical signals that can drive nonspecific cell behavior. Furthermore, the easy availability of autologous PL and their combination with patient‐derived cells can open up the possibility of applying PLMA‐based hydrogels as a 3D platform for personalized drug screening.

In this study, we introduced the aforementioned new human‐based biomimetic material, PLMA hydrogels, as 3D platforms for cell spheroid invasiveness studies. In order to evaluate the applicability of PLMA hydrogels for this purpose, rBM of murine Engelbreth‐Holm‐Swarm sarcoma (Matrigel) was used as a positive control since, besides to be considered the “gold standard” for 3D in vitro disease models development, it is also constituted by a complex mixture of proteins. Spheroids of three tumor cell lines (osteosarcoma, MG‐63 and SaOS‐2, and lung cancer, A549) as well as human bone‐marrow mesenchymal stem cells (hBM‐MSC) were embedded into PLMA hydrogels at three different concentrations, Matrigel and poly(ethylene glycol) diacrylate (PEGDA), the latter used as negative control. The easy control over the mechanical properties of PLMA hydrogels offer the opportunity of evaluating the matrix conditions that more closely reproduce the micromechanical environment depending on the tissue under study. In this sense, the invasiveness of each cell type was systematically analyzed with the aim of determining the invasion kinetics and uniformity, and evaluate the PLMA hydrogels mechanical conditions that potentiate the best approximation to in vivo cell invasiveness behavior.

## 2 Results and Discussion

Advances in 3D in vitro systems have been contributed to the recreation of the cellular heterogeneity and microenvironment dynamism of several tissues, providing robust platforms with potential to be applied in preclinical drug screening studies. Although the cell–ECM interaction is considered as crucial in physiological and pathological events, most of the 3D in vitro models are still based on multicellular tissue spheroids (MCTS) cultured in a scaffold‐free setting.^14^, ^28^ However, regarding some pathologies as cancer, the understanding of how a growing tumor interacts with the ECM, invades through it and responds to chemotherapeutic treatments into this dynamic environment can expedite therapy development.^24^, ^37^ To date, only a few studies have combined scaffold‐free spheroids with ECM‐resembling scaffolds. Nevertheless, the importance of a surrounding matrix in the recapitulation of cell–cell and cell–ECM interaction during early metastatic processes has been demonstrated, evidencing spheroid embedding into a matrix as a promising strategy to assess MCTS growth, invasiveness ability, and antimetastatic drug sensitivity.^38^, ^39^

**Figure 1:**
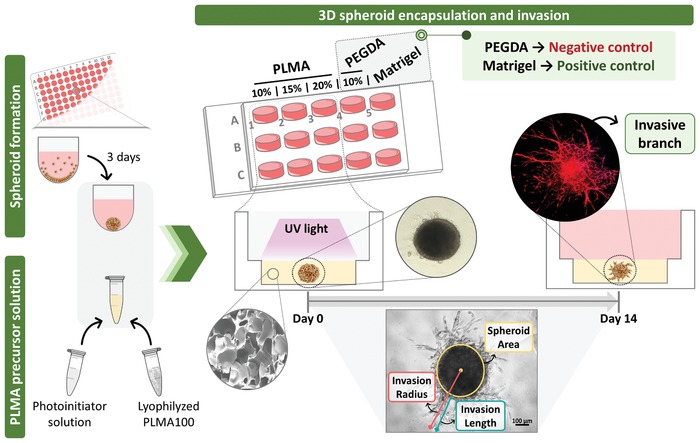
Schematics of 3D spheroids encapsulation to study cell spheroid invasiveness. Spheroids are formed into round‐bottom ultralow attachment plates during 3 d. The PLMA precursor solution is obtained solubilizing lyophilized PLMA in a photoinitiator solution for UV photopolymerization. Generated spheroids are encapsulated into PLMA hydrogels at three different concentrations (10, 15, and 20% (w/v)), poly(ethylene glycol) diacrylate (PEGDA) at 10% (w/v), and Matrigel. Over the time of culture, the encapsulated cells start to invade the surrounding ECM‐mimicking matrix, developing the named invasive branches. Throughout the 14 d of culture, the spheroid area and invasion length are measured to evaluate the invasiveness ability of each cell type into the different biomaterials and stiffnesses.

In an attempt to provide a reliable platform for 3D spheroid invasion, overcoming the recognized limitations of the currently applied biomaterials, we herein explore for the first time the potential of a newer photoresponsive humanized material, the PLMA hydrogels, to support MCTS growth and invasion (**Figure** **1**). Spheroids of stem cells (hBM‐MSC) and cancer cell lines (MG‐63, SaOS‐2, and A549) were embedded into PLMA hydrogels. The human origin and tunable mechanical cues of these hydrogels are expected to produce a more faithfully recapitulation of human cellular behavior, supporting the individual invasiveness ability of each cell line.^33^ Osteosarcoma and lung cancer cell lines were chosen in order to demonstrate that PLMA hydrogels are able to sustain the growth of malignant tumors of mesenchymal and epithelial origin, respectively.^40^ Although tumor models are a research hotspot, the role of mesenchymal stem cells (MSC) on fundamental physiological and pathological events has also been a topic of increasing interest on biomedical field. The aggregation of MSC into spheroids has been suggested as an improvement of stem cell‐based therapies once the enhancement of anti‐inflammatory and angiogenic properties, stemness and survival of MSC have been reported in this 3D configuration.^41^, ^42^, ^43^ In this sense, the ability of PLMA hydrogels to support hBM‐MSC cell culture in a spheroid setting was also evaluated.

### 2.1 Uniformity of Spheroid Formation

Matrix‐dependent cell spheroid invasion should be evaluated assuming the same conditions in terms of initial spheroid size for each cell type in order to assure accurate and reproducible results. Hanging drop and forced‐floating are robust scaffold‐free techniques to spheroid generation which are expected to yield tunable and reproducible spheroids with normally distribution of initial sizes.^6^, ^44^ In this study, 96‐well round‐bottom ultralow attachment plates were used to generate spheroids of four cell types (hBM‐MSC, MG‐63, SaOS‐2, and A549). An optimal seeding density of 12 000 cells/spheroid was established to produce spheroids with a mean size of 300–600 µm in diameter at 72 h, promoting the establishment of a cell heterogeneity suitable for physiopathological studies.

hBM‐MSCs and MG‐63 are cell types characterized by the production of a dense ECM and expression of cell‐mediated adhesion proteins, which increase the spontaneous aggregation ability that results into highly compact and circular spheroids (Figure S1A,B, Supporting Information). However, not all cell types demonstrate this ability, with nonspheroid forming cells producing loose aggregates as a result of weak intercellular interactions (Figure S1C,D, Supporting Information).^45^ The lack of spheroid‐forming ability is related to the poor expression levels of cadherin and catenin protein families, which are involved in cell–cell adhesion via adherens junctions.^46^, ^47^ To overcome this barrier in spheroid formation, biological and synthetic additives acting as cross‐linking agents,^48^, ^49^ adhesion stimulators,^48^, ^50^ or rheological modifiers and crowding agents^51^, ^52^ have been applied to strengthen cellular aggregation. Herein, methylcellulose (MethoCel) was used as crowding agent to improve the spheroid formation from SaOS‐2 and A549 cell lines, leveraging its semisolid gel‐like properties that increase the culture medium viscosity. The supplementation with MethoCel strengthened cell–cell interactions, allowing the formation of stable but not highly compacted SaOS‐2 and A549 aggregates after 3 d of formation (Figure S1C,D, Supporting Information). Concerning to the area of the spheroids generated from each cell type, hBM‐MSC and MG‐63 spheroids are the smallest ((1.12 ± 0.211) × 10^5^ and (1.20 ± 0.104) × 10^5^ µm^2^, respectively) due to the strongest intercellular interaction that produced compacted spheroids—see **Table** **1** and Figure S1A,B (Supporting Information). In SaOS‐2 and A549 spheroids, the cells were not highly compacted, resulting in a higher average size ((3.19 ± 0.289) × 10^5^ µm^2^ for SaOS‐2, and (3.34 ± 0.273) × 10^5^ µm^2^ for A549)—see Table 1 and Figure S1C,D (Supporting Information). Analysis of size distribution suggests that spheroids were uniformly generated producing a Gaussian distribution of their initial sizes, as confirmed by D'Agostino–Pearson normality test (Figure S2, Supporting Information).

**Table 1:** Area and diameter of the four cell spheroids types. Spheroids were obtained from 12 000 cells cultured during 3 d on round‐bottom ultralow attachment plates. Spheroid diameter was obtained through spheroid area data considering the spheroids as perfect circumferences

Cell type	Spheroid area [× 10^5^ µm^2^]	Spheroid diameter [µm]
hBM‐MSC	1.12 ± 0.21	376.30 ± 35.57
MG‐63	1.20 ± 0.10	390.85 ± 16.61
SaOS‐2	3.19 ± 0.29	637.05 ± 28.61
A549	3.34 ± 0.27	651.78 ± 26.32

### 2.2 3D Embedded Spheroid Culture and Morphological Characterization

Collective cell movement into the surrounding tissues plays a crucial role in morphogenesis, physiological processes (e.g., wound healing), and also in cancer spreading and metastasis development.^22^ Several evidences have demonstrated that, in addition to cell‐intrinsic mechanisms, biochemical and mechanical cues of the ECM are fundamental factors in cell invasion.^29^, ^53^ From this standpoint, recapitulation of the pathological processes triggered by the microenvironment components could elucidate about the involved molecular mechanisms and drive the development of new therapeutic targets.

**Figure 2:**
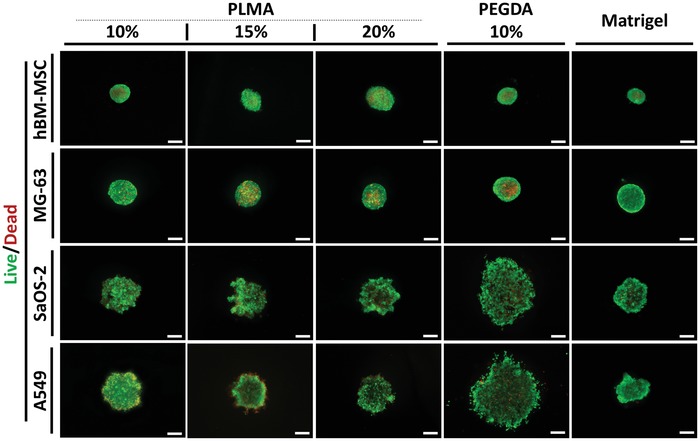
Fluorescence microscopy images of Live/Dead staining. hBM‐MSC, MG‐63, SaOS‐2, and A549 spheroids encapsulated into PLMA hydrogel (10, 15, and 20% (w/v)), PEGDA hydrogel (10% (w/v)), and Matrigel at 24 h post encapsulation. All spheroids were generated with an initial number of 12 000 cells per spheroid. The green and red channels represent the Calcein‐AM and PI staining of live and dead cells, respectively. Scale bar: 200 µm.

**Figure 3:**
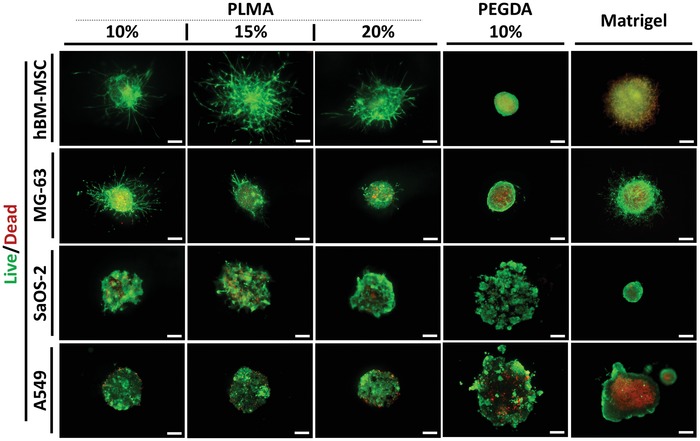
Fluorescence microscopy images of Live/Dead staining. hBM‐MSC, MG‐63, SaOS‐2, and A549 spheroids encapsulated into PLMA hydrogel (10, 15, and 20% (w/v)), PEGDA hydrogel (10% (w/v)), and Matrigel at 14 d of culture. All spheroids were generated with an initial number of 12 000 cells per spheroid. The green and red channels represent the Calcein‐AM and PI staining of live and dead cells, respectively. Scale bar: 200 µm.

**Figure 4:**
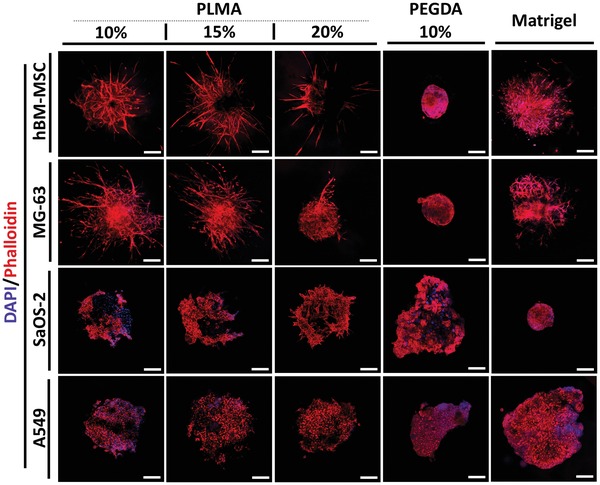
Fluorescence microscopy images of DAPI/phalloidin staining. hBM‐MSC, MG‐63, SaOS‐2, and A549 spheroids encapsulated into PLMA hydrogel (10, 15, and 20% (w/v)), PEGDA hydrogel (10% (w/v)), and Matrigel at 14 d of culture. All spheroids were generated with an initial number of 12 000 cells per spheroid. The blue channel represents the nuclear staining by DAPI and the red channel demonstrates the actin filaments staining by Phalloidin‐Red probe. Scale bar: 200 µm.

rBM and type I collagen‐based invasion models have been widely used in invasion‐related studies.^54^, ^55^ PLMA‐based hydrogels were recently proposed as a new biomimetic material with tunable mechanical properties for 3D cell culture.^33^ Herein, we aimed to demonstrate that PLMA hydrogels are also suitable to sustain spheroid heterogeneity and support cell spheroid invasion. To validate the cell spheroid behavior into PLMA hydrogels, Matrigel and PEGDA were chosen as positive and negative controls, respectively. The choice of Matrigel instead of type I collagen is related with the fact that Matrigel is characterized by a complex mixture of proteins, thus more closely resembling the composition of PLMA. Regarding the negative control, PEGDA is a photopolymerizable synthetic polymer via UV light exposure, such as PLMA, but its lack of adhesive motifs makes it unable to support cell invasion. In order to explore the effect of the mechanical properties on spheroid behavior and cell invasiveness, compressive mechanical tests were performed in PLMA hydrogels at 10, 15, and 20% (w/v), and the PEGDA hydrogel concentration was chosen to have similar mechanical properties (Figure S3, Supporting Information). The PLMA hydrogels herein explored have a Young's modulus in the range of 13–16 kPa and the PEGDA hydrogel at 10% (w/v) has a Young's modulus of 12 kPa. Regarding the mechanical properties of Matrigel, Davidson et al.^56^ reported a Young's modulus of 401 Pa obtained through a compression test. For this study, a 14 d invasion assay was carried out on the encapsulated spheroids generated from the four types of cells already mentioned in the different types and concentrations of biomaterials (Figures S4–S7, Supporting Information). Cells in spheroids with a diameter range from 200 to 500 µm start to develop chemical gradients (e.g., nutrients, oxygen, pH) that define a typical zone of proliferative cells at the surface and necrotic/hypoxic cells in the core, sometimes coexisting with quiescent cells in the middle.^19^ In order to analyze this heterogeneity, Live/Dead assays were performed using Calcein‐AM and PI staining for all conditions (cell spheroid type vs biomaterial type/concentration). After 24 h of spheroid encapsulation, fluorescence microscopy evidenced that hBM‐MSC and MG‐63 spheroids presented a well‐defined necrotic core surrounded by a zone of metabolically active cells (**Figure** **2**). Effectively, the compacted structure of these two types of spheroids as well as their sizes is favorable for the development of this in vivo‐like phenotypic heterogeneity. At the end of the 14 d of culture, hBM‐MSC and MG‐63 showed high cell viability and invasion for all concentrations of PLMA hydrogels—see **Figures** **3** and **4**. In the case of hBM‐MSC spheroids, the necrotic core is significantly smaller in comparison with tumor‐derived spheroids, correlating with the study developed by Bartosh et al.,^41^ where the authors showed that only a reduced fraction of cells are apoptotic or necrotic in a hBM‐MSC spheroid culture. Spheroids embedded into PEGDA hydrogels maintained an outer ring of viable cells surrounding a well‐defined necrotic core (Figure 3). When embedded into Matrigel, MG‐63 spheroids with an evident necrotic core invaded the hydrogel, whereas cell–ECM interaction seemed to promote mesenchymal cells invasion in hBM‐MSC spheroids. However, the invading hBM‐MSCs undergone in a quiescent state, which is demonstrated by the overlap of red and green stains (Figure 3). The entry of MSCs into a quiescent state is known to be related with loose cell adhesion due to anchorage deprivation.^57^, ^58^, ^59^ So, the cell behavior herein showed by Matrigel‐embedded hBM‐MSC spheroids was triggered by scaffold softness, an important physicomechanical cue recently explored by Rumman et al.^59^ using polyacrylamide gels of varying stiffness to induce cells quiescence. Although this cellular state is interesting to cell differentiation, cell invasion studies must be conducted using an ECM‐resembling matrix with improved mechanical properties able to support the viability of invasive cells. Regarding this issue, PLMA hydrogels clearly demonstrated to support hBM‐MSC viability, offering the proper mechanical cues to allow the formation of robust actin stress fibers and focal adhesions, contrary to Matrigel (Figure 4).

**Figure 5:**
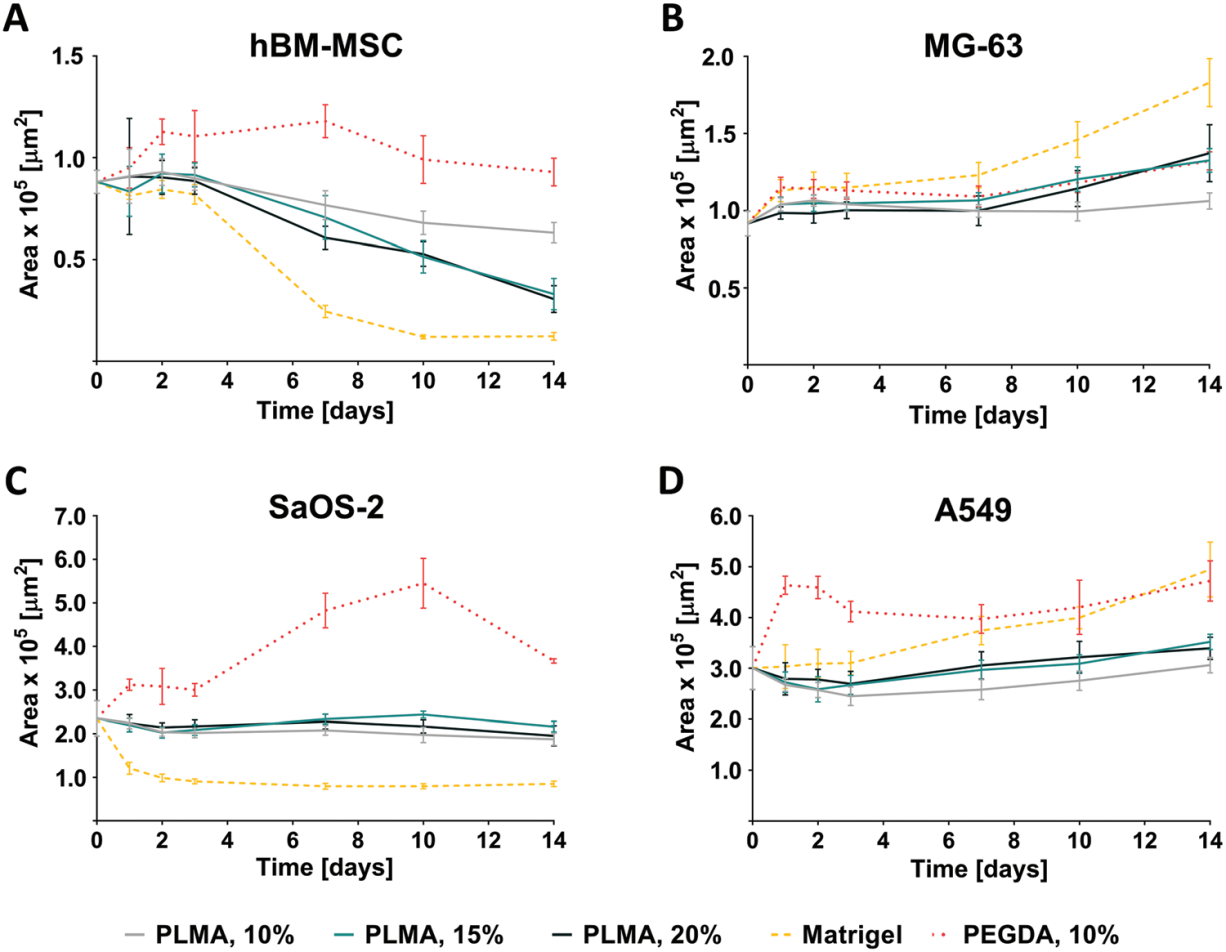
Progression of spheroids area during the 14 d of culture. A–D) Quantification of the area of hBM‐MSC, MG‐63, SaOS‐2, and A549 spheroids into the different biomaterials: PLMA hydrogel (10, 15, and 20% (w/v)), PEGDA hydrogel (10% (w/v)), and Matrigel. Data are presented as mean ± SD (*n* ≥ 3).

With regard to SaOS‐2 spheroids, the above‐discussed issues concerning the formation of noncompacted cellular aggregates resulted in a homogeneous distribution of live and dead cells in those spheroids at 24 h of encapsulation (Figure 2). Over the 14 d of culture, the cells freely organized inside the PLMA hydrogel and Matrigel, forming a necrotic core, what was not verified into the negative control, PEGDA hydrogels. The homogeneous distribution of live and dead cells at 24 h of encapsulation was also verified in A549 spheroids encapsulated into the three biomaterials (Figure 2). However, in A549 spheroid embedded into PLMA hydrogels, an outer ring of dead cells was observed. It can be explained with the occurrence of solid stress imposed by the surrounding ECM.^60^, ^61^, ^62^ This mechanical compressive stress can induce genotypic and phenotypic changes associated with a higher tumor malignancy that can be reflected into an increased tumor invasiveness ability.^62^ On the other hand, this physical constraint can affect cell viability and promote apoptosis, an event that is reported to be reversible.^61^, ^63^ Effectively, the elastic modulus of PLMA hydrogels (13–16 kPa) is much higher than Matrigel (401 Pa), what means that the compressive stress applied by PLMA hydrogels is quite higher (Figure S3, Supporting Information).^33^, ^56^ Cheng et al.^61^ demonstrated that when an external stress (2 kPa) is applied on agarose‐encapsulated spheroids, the caspase‐3 activity increases. From spheroid area analysis, it is observable that A549 spheroid significantly compacted into PLMA hydrogels during the first 24 h post encapsulation, which demonstrate the compressive stress exerted by this PL‐derived matrix that drive cell death (**Figure** **5**). In this mechanical context, PLMA‐encapsulated A549 spheroids are able to overcome this inhibitory feedback exerted by the surrounding matrix once PLMA is a protein‐based hydrogel and, for that reason, can be degraded and softened by released matrix metalloproteinases. After 14 d of culture, the presence of dead cells in the periphery was still verified, but to a lesser extent (Figure 3). These results demonstrate that the cells were able to overcome the described negative effect of the ECM compressive stress. Contrary to that described for SaOS‐2, A549 spheroids did not compact into Matrigel and were able to develop a highly defined necrotic core. In PEGDA, the majority of the cells were still viable and dead cells were mostly located in the center of the spheroid (Figure 3).

4′,6‐Diamidino‐2‐phenylindole (DAPI)/phalloidin staining was performed at 14 d of culture in order to analyze the morphology of actin filaments, which are involved in the regulation of cell shape and polarity, playing a crucial role in cell motility. Fluorescence images obtained from z‐stacks show that invading cells from hBM‐MSC and MG‐63 spheroids acquired an in vivo‐like cell polarity in PLMA hydrogels and Matrigel—see Figure 4. Nevertheless, the pattern of invasion is clearly distinct, with a more evident collective invasion pattern into PLMA‐based matrix contrasting with a higher number of cells individually migrating into Matrigel. Moreover, the actin fibers stress is strongest in PLMA hydrogel, as can be seen by the most pronounced cell elongation comparing with cells invading Matrigel. hBM‐MSC spheroids presented an open spheroid structure due to the interconnectivity between collective invading cells. MG‐63 spheroids showed to maintain their compact structure (denser zones), surrounded by an interconnective network of tumor invading cells. These structural features were not verified in Matrigel‐embedded spheroids, where the cells that constitute the spheroid dispersed inside the matrix without defined invasive branches (Figure 4).

In SaOS‐2 and A549, the intercellular interactions between aggregated cells embedded into PLMA hydrogels are visible as well as some small invasions (Figure 4). A549 cell spheroids demonstrated to self‐organize and form round to oval‐shaped noncellular regions in PLMA hydrogels and Matrigel, also visible in Live/Dead fluorescence images (Figure 3) that, according to in vivo histology analysis in literature, corresponds to malignant glands of acinar adenocarcinoma with invasive phenotype.^64^ It clearly evidences that PLMA hydrogel stimulates an in vivo‐like cell organization of lung cancer cells and promotes an invasive behavior. To the best of our knowledge, this is the first time that this invasive phenotype was recapitulated in a spheroid‐based 3D in vitro culture, what can be useful to perform pathological studies in a more realistic way. This different tumor cell behavior into Matrigel is probably related with the matrix stiffness since lung cells are originated from a soft environment, then the environment provided by Matrigel is mechanically closest to the native one.

With regard to cell proliferation, ATP quantification indicates a decrease of this proliferative marker over 14 d in all cell types, except in MG‐63 (Figure S8, Supporting Information). ATP production is dependent on each cell energy demand and is related to the metabolic pathways adopted by cells to trigger specific pathophysiological processes. Some studies reporting the use of ECM‐mimicking matrices to embed cell spheroids have been demonstrating that the matrix can exert a compressive solid stress responsible for changes in gene expression related with cell proliferation, apoptosis, invasion, and migration.^60^, ^63^, ^65^ That mechanical stress stimulates the expression of proliferation inhibitors, inducing cell‐cycle arrest, and was also demonstrated to drive cell phenotype switching between proliferative and invasive behavior.^62^, ^63^ Effectively, some reports have evidenced the hypothesis of phenotype switching as a key event characterized by cell‐cycle arrest involved not only in cancer metastasis, but also in physiological processes as embryogenesis.^66^, ^67^ These two central phenotypes in malignant behavior were already studied in vitro using Matrigel invasion assays^68^, ^69^ and confirmed in vivo by immunohistochemistry of invasive and proliferative cells of patient tumor samples.^70^, ^71^ The decrease of ATP in hBM‐MSCs, SaOS‐2, and A549 spheroids can be explained by the switching from proliferative to invasive phenotype. As expected, in PEGDA hydrogels, due to the inexistence of proteolytic degradation, no cell type was able to invade, however the compressive stress may also have an effect on proliferation inhibition. Concerning the increased ATP production of MG‐63 from 24 h to 14 d of culture (Figure S8, Supporting Information), we hypothesize that this cell line is more resistant to mechanical stress than other cells. Moreover, the most pronounced ATP increase in protein‐derived matrices can be related with a high expression of metalloproteinases (MMPs) comparing with other cells, as SaOS‐2.^72^

Concerning the ability of PLMA hydrogels to sustain the formation of a necrotic core surrounded by an outer zone of viable cells, the results indicate that 3D tumor spheroids of MG‐63 and SaOS‐2 embedded into this matrix recapitulate the in vivo‐like phenotypic heterogeneity of solid tumors (Figure 3). In the case of hBM‐MSC, this heterogeneity was also verified, demonstrating the feasibility of PLMA hydrogels to support MSC spheroid culture and explore this approach for MSC‐based therapeutics research.^73^ Although A549 spheroids did not develop a necrotic core, optical contrast and fluorescence microscopy at 14 d of culture demonstrated that the cells were able to self‐organize and develop acinar structures particularly related with an invasive phenotype, validating the applicability of PLMA hydrogels for spheroid‐based lung cancer‐related studies. As expected, the four cell spheroid types encapsulated into PEGDA hydrogels were not able to invade the matrix, and significant structural changes were not visualized.

### 2.3 Matrix‐Dependent 3D Spheroid Area and Invasion Speed

Besides the evaluation of the spheroid phenotypic heterogeneity and invasive cells morphology, the consistency of invasion is a prerequisite to the development of accurate and reproducible in vitro invasion models.^44^ Therefore, spheroid core area throughout the invasion assay and uniformity of invasion of each cell spheroid type into the different matrices were assessed through optical microscopy images analysis. For spheroid area analysis, it is important to note that only the area corresponding to regions where the spheroid cells were compact was considered in order to investigate the compact spheroid area maintenance—see Figure 1.

Spheroid area changes during the 14 d of culture were visually distinct between each cell type and highly dependent on the biomaterial where they were embedded (Figure 5). In hBM‐MSC spheroids, a diminishing in their area was verified into PLMA hydrogels and Matrigel as a result of the mesenchymal‐related invasiveness ability (Figure 5A). Regarding their behavior into PLMA hydrogels of different stiffness, the area suffered a further decrease on the stiffest hydrogels (15% and 20% (w/v) of PLMA). Nonetheless, a more pronounced decrease in the area of the spheroid core was seen in Matrigel, what is related with the aforediscussed dispersity of the cells that constitute the compact area of the spheroid. In PEGDA, despite some oscillations during the culture time, the difference between the initial and final area was not significant. For MG‐63 spheroids, no significant changes in area values were verified when encapsulated into PLMA or PEGDA hydrogels (Figure 5B). In Matrigel, an increase in spheroid area was observed, as clearly evidenced by 24 h and 14 d Live/Dead images (Figure 3). SaOS‐2 spheroids embedded into PLMA hydrogels remained confined over the 14 d with a minimal decrease in the area value, which is probably related with the maintenance of cell adhesion proteins expression (Figure 5C). It demonstrates that the herein proposed new biomaterial is not stimulating alterations on the gene expression levels of this cell type. Into PEGDA hydrogels, the cell aggregates seem to have dispersed during the first 7 d; after that, the weak cell–cell interactions were enough to promote cell reaggregation, being initial and final spheroid sizes similar. These spheroids demonstrated to have a different behavior when encapsulated into Matrigel, where the cells quickly compacted as seen in Figure 4. Contrary to what was observed in PLMA‐based hydrogels, the very weak cell–ECM interactions along with an improved cell–cell interaction via E‐cadherin expression can explain this particular behavior. Effectively, it is well‐reported that the stiffening of the tumor‐surrounding ECM is highly related with an invasive and metastatic phenotype, where cell–ECM interaction is improved.^29^, ^74^, ^75^, ^76^ It means that the softness of Matrigel can probably be inducing increased E‐cadherin and decreased N‐cadherin expression, epithelial and mesenchymal‐associated markers, respectively.^76^, ^77^ Relatively to A549, the other cell line classified as nonspheroid forming cells, cell aggregates compaction in PLMA hydrogels was observed during the first 2–3 d of culture, what can be related with the compressive force exerted by the surrounding matrix that led to cell death on the periphery of the spheroids—see Figure 5D. Then, in accordance with the enhanced cell viability until 14 d, spheroid area significantly increased. When embedded into Matrigel, A549 spheroid area increased over the 14 d. This limited growth into PLMA hydrogels in comparison with Matrigel is related with the mechanical stress imposed by the higher stiffness of PLMA matrix (Figure S3, Supporting Information).^60^, ^63^ In PEGDA hydrogels, A549 cells aggregates firstly dispersed inside the gel. Perhaps due to the lack of adhesion motifs in PEGDA network, the cells compacted in a spheroid structure where cell–cell adhesion prevailed.

Altogether, the spheroid area throughout the invasion assay showed to be highly dependent on the biomaterial and cell type. Nevertheless, the spheroid area tendency demonstrated that PLMA hydrogels guarantee a spheroid behavior similar to verified into Matrigel and PEGDA hydrogels, evidencing the potential of this new humanized biomaterial for further fundamental and therapeutic studies. Furthermore, the matrix stiffness also seemed to contribute to this parameter but in a lesser extent, demonstrating that matrix biochemical features are more important with regard to changes in spheroids area. In this respect, PLMA hydrogels are evidently the currently available biomaterial that offer the matrix biochemical cues to better recapitulate cell behavior.

**Figure 6:**
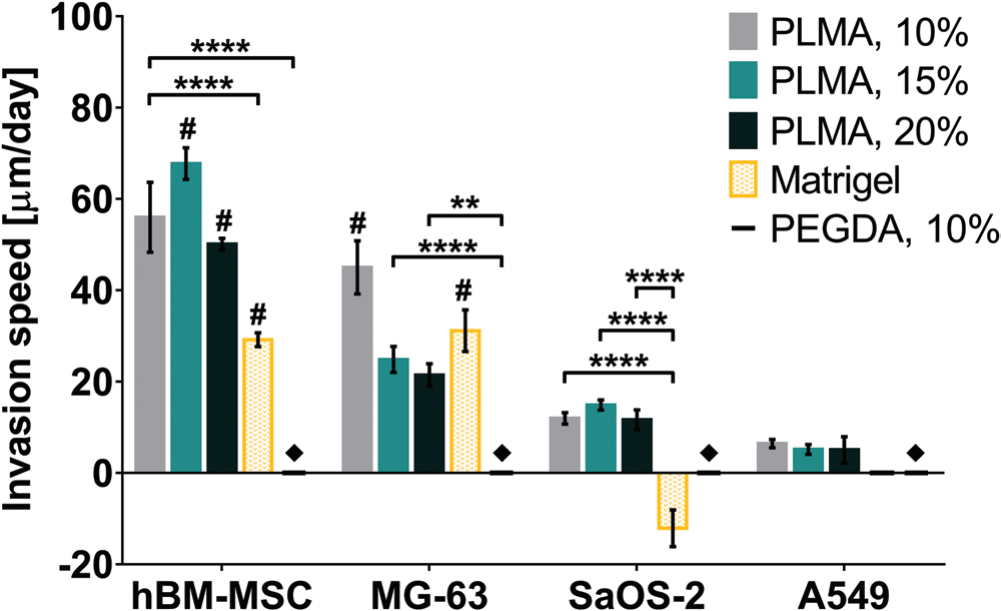
Invasion speed of each spheroid type into the different biomaterials and stiffness conditions. Data results from a linear regression analysis of the invasion length up to 14 d of culture and are presented as mean ± SD (*n* ≥ 3). Statistical analysis was performed between the different biomaterials used in each cell spheroid type. Two‐way ANOVA analysis of variance combined with Tukey's multiple comparisons test revealed significant differences between analyzed groups: **p < 0.01 and ****p < 0.0001. **#** means significant differences with the groups on the right, inside the same cell spheroid type group. For the remaining groups, the statistical analysis revealed no significant differences. ♦ PEGDA‐embedded spheroid did not invade the hydrogels.

In terms of invasion kinetics of each cell spheroid type into a matrix, it is important to develop a platform that could sustain a uniform invasion, ensuring reproducibility and correctness of the results. To explore this issue in the PLMA matrix‐based models, the invasion length of the spheroids was measured during the first 72 h and at 7, 10, and 14 d of culture, and compared with the results obtained on the negative and positive controls. The invasion length was analyzed, and the invasion speed was determined through a linear regression of the curves of invasion length versus time—see Figure S9 (Supporting Information). The invasion speed for each condition was expressed as mean ± standard deviation (SD) and the uniformity of invasion was analyzed recording the goodness of fit (*R* ^2^) through the above‐referred linear regression—see **Figure** **6** and Table S1 (Supporting Information). We assumed that *R* ^2^ ≥ 0.80 represents the range where the invasion is considered sufficiently uniform to validate the correspondent condition as an accurate and robust invasion model. Figure 6 resumes the invasion speed of the different cells cultured in the three hydrogels, considering the distinct stiffness conditions of PLMA hydrogels.

As expected, into the hydrogel considered as the negative control, PEGDA, the lack of cellular adhesion motifs and domains sensitive to cell‐mediated proteolysis hindered cell invasion of all cell types studied here—see Figure 6. The two cell types with higher spheroid‐forming ability, hBM‐MSC and MG‐63, also demonstrated the greatest invasiveness capacity in PLMA‐ and Matrigel‐based hydrogels. hBM‐MSC showed to have a higher invasion kinetics in the three PLMA formulations than in Matrigel, displaying variability of invasion speed between distinct stiffnesses of PLMA. Regarding MG‐63 cells, different invasion capacity was also verified but, in this case, only the PLMA at 10% (w/v) showed to enhance the invasion kinetics in comparison to Matrigel. Although MG‐63 cells exhibit mesenchymal features as hBM‐MSC, the data from PLMA biomaterial show that this cell type prefer softer matrices than mesenchymal stem cells.

Some reports in which invasion and migration of SaOS‐2 and MG‐63 were evaluated into Transwell chambers, both cell lines exhibited similar ability regarding these parameters.^78^, ^79^, ^80^ However, the herein presented data suggest that these two osteosarcoma cell lines have a significantly different behavior of invasion. As showed in Figure 6 and unlike to what is described in literature, SaOS‐2 demonstrated to have less invasive ability in all PLMA concentrations comparing with MG‐63. It is probably related with the fact that cell invasion has been evaluated throughout an embedded cell aggregate, spheroid, and not as cell suspension seeded on a matrix. Besides that, it is reported that spheroid compaction is associated with an aggressive invasive phenotype,^81^ which correlates with the presented data once SaOS‐2 formed noncompacted cell aggregates contrary to MG‐63 cells. Nonetheless, once spheroid is considered the most relevant 3D platform, cell invasion studies could be more accurate when evaluated through a cell spheroid due to the tridimensionality that more faithfully mimics an in vivo tumor invasion. In Matrigel^®^, the value of SaOS‐2 invasion kinetics was negative, which is related with the huge spheroid compaction observed during the first hours (Figure 5B). This means that, although small invasions have been visualized and were similar to the ones observed in PLMA hydrogels (Figure S6, Supporting Information), the radius of invasion was smaller than the radius of the spheroid measured at day 0.

A549 is an epithelial cell line able to form primary tumors and pulmonary metastasis into immunocompromised mice.^82^ However, 3D invasion models to recapitulate and study the metastatic process are not well‐explored. So, we intended to investigate the ability of A549 cells to invade throughout an PLMA‐embedded spheroid. Interestingly, A549 were able to create small protrusions in PLMA, contrary to what was verified in Matrigel, where the cells were not able to invade in the matrix—see Figure 6. Although the low invasion speed, the data suggest that PLMA‐based hydrogels can sustain A549 invasion, offering a 3D platform to develop a lung invasion model to more faithfully study pulmonary metastasis. To the best of our knowledge, this is the first time that spheroid‐forming A549 cells demonstrated the ability to invade an involving matrix, even in a small extension, in the absence of stromal cells. Some studies have reported the importance of stromal cells (e.g., endothelial cells) into this metastatic process of A549 and established the key role of growth factors, such as epidermal growth factor (EGF) and transforming growth factor beta 1 (TGF‐β1), in epithelial–mesenchymal transition (EMT).^83^, ^84^ Taking into account the abundance of growth factors present in the raw material of the hydrogels here proposed, PLMA hydrogels are probably triggering the EMT of A549, stimulating its invasion through the matrix.^33^

The goodness of fit (*R* ^2^) obtained through the linear regression to determine the invasion kinetics is a useful parameter to analyze how uniformly the spheroids invade into the distinct matrices. This parameter, along with the value of invasion kinetics, is essential to choose the matrix whose biochemical and mechanical properties offer the best condition to support an in vivo‐like invasion of a cell type and, by this way, develop a faithful invasion model. In general, the results show that PLMA hydrogels support a uniform invasion of the four cell types. Only the invasion of A549 spheroids into PLMA at 20% (w/v) cannot be considered uniform. Regarding the spheroid invasiveness into Matrigel, hBM‐MSC and MG‐63 spheroids were able to invade uniformly, although it was found that hBM‐MSC entered in a quiescent state. The same does not hold true for SaOS‐2 spheroids, for which *R* ^2^ < 0.80.

Overall, PLMA hydrogels demonstrate to support the invasiveness ability and uniformity of the different spheroids in comparison to positive control, Matrigel. The data also show that the invasion kinetics can be controlled through hydrogel stiffness, demonstrating the ease of adapting PLMA hydrogels to the biomechanical cues of the native tissue under study.

### 2.4 Growth Factors Release from PLMA Hydrogels

Platelet lysates (PL) are protein concentrates rich in fibrous and adhesive proteins, growth factors, and other bioactive molecules that play a pivotal role in physiological processes, such as wound healing and tissue regeneration.^34^ Although the proteins undergo a chemical modification process, the degree of modification is about 14%, allowing a sustained release of proteins from the PLMA hydrogel matrices, as previously reported.^33^ Hence, protein releasing from PLMA hydrogels at 10, 15, and 20% (w/v) was herein performed in order to evaluate the release of VEGF‐A, TGF‐β1, and EGF. These growth factors have been associated with the induction of EMT and, by this way, related with an increased cell invasiveness and metastatic dissemination in primary tumor sites.^85^, ^86^, ^87^, ^88^ In this sense, the assessment of their presence and release in the PLMA‐based invasion models is an important step toward the validation of this human‐derived matrix for 3D invasion models application.

**Figure 7:**
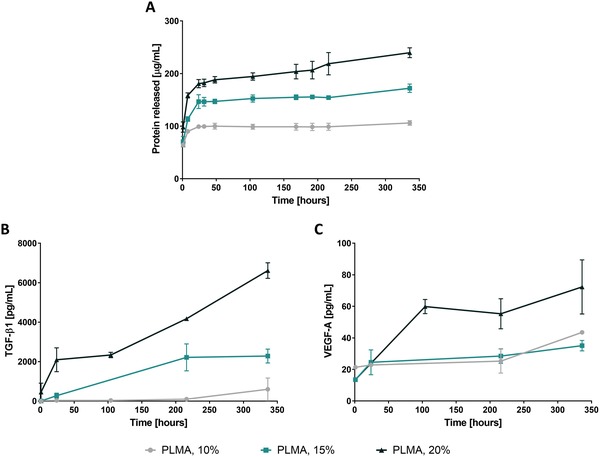
Protein and growth factor release from PLMA hydrogels. A) Total protein quantification and B,C) ELISA quantification of TGF‐β1 and VEGF‐A release from PLMA hydrogels at 10, 15, and 20% (w/v). Data are presented as mean ± SD (*n* ≥ 3).

The release profile of total protein showed a fast release during the first 24 h, followed by a sustained release until the 336 h (14 d) (**Figure** **7**A). This tendency is in accordance with the previously reported analysis.^33^ To address the release of the aforementioned growth factors, ELISA assays were performed and a sustained release of VEGF‐A and TGF‐β1 was verified (Figure 7B,C). As expected, VEGF‐A and TGF‐β1 ELISA quantification assays suggest a dependency of the protein released concentration relatively to the PLMA hydrogels concentration. Comparing with the availability of growth factors reported for standard Corning Matrigel, VEGF‐A and TGF‐β1 release from PLMA hydrogels is until 100‐fold lower and 2‐fold higher, respectively. EGF ELISA quantification revealed that this growth factor was not released from PLMA hydrogels in its ELISA‐detectable form, while in standard Corning Matrigel its presence is reported. One of the reasons that may justify the nondetection of this growth factor is its loss during the dialysis process, since its molecular weight (≈6.2 kDa) is slightly higher than the cutoff (3.5–5.0 kDa) of the dialysis membranes used. On the other hand, EGF may have undergone chemical modification at the ELISA recognition site; however, its release in a biologically active form may be occurring. Furthermore, matrix‐immobilized growth factors can also display available biologically active domains to directly interact with encapsulated cells and induce in vivo‐like cell behaviors. In this sense, for any of the growth factors here quantified, it can be hypothesized that their availability is higher than that detected in the ELISA assays. From this standpoint, the protein content availability of PLMA hydrogels reinforces the hypothesis that their biochemical composition recreates the protein accessibility in the native tissues ECM, promoting the exceptional cell invasiveness behavior herein reported.

## 3 Conclusions

The demand for more pathophysiologically relevant 3D models able to recapitulate fundamental features of tumor metastasis is ever‐rising in order to further improve the discovery of new effective therapies. PLMA‐based hydrogels, recently proposed as a new biomaterial for 3D cell culture, were herein explored as a human‐derived platform for the development of spheroid invasion models. This biomaterial demonstrated to support cellular invasion and formation of a necrotic core in spheroids of hBM‐MSC and distinct tumor cell lines, recreating the phenotypic heterogeneity of solid tumors which is undoubtedly involved in therapy resistance in vivo. Furthermore, the cells also acquired an in vivo‐like cell polarity and were able to invade the matrix, forming complex interconnective cellular networks. Regarding the invasiveness ability evaluation of each cell spheroid type, PLMA‐based hydrogels not only promoted an increased invasion kinetics comparing with the positive control, Matrigel, but also demonstrated that the invasion kinetics can be controlled through PLMA matrix stiffness. We hypothesized that this invasion kinetics can be associated with the release or cell–ECM interaction of crucial growth factors (e.g., VEGF, TGF‐β1, EGF, IGF) involved into the stimulation of cellular invasion and migration during tumor growth and metastasis development.

Overall, these results clearly suggest that PLMA‐based hydrogels can be a great alternative to the animal‐derived Matrigel. The proposed 3D invasion models may be used to study the biological mechanisms involved in metastatic cascade and as a platform for screening and validation of new therapeutic agents in a more realistic microenvironment. The evidenced ability to support different cell population survival also opens up the possibility to develop more complex disease models, integrating and studying the role of stromal cells in proliferation and drug resistance of tumor cells. Finally, the possibility of combining patient‐derived PL and cells can allow the application of this platform in personalized drug screening.

## 4 Experimental Section

##### Synthesis of Methacryloyl Platelet Lysates

PLMA were synthesized based on a protocol previously reported.^33^ Briefly, PL (STEMCELL Technologies, Canada) were thawed in a water bath at 37 °C. Then, PLMA of low‐degree modification (PLMA100) were synthesized by reaction with methacrylic anhydride (MA) 94% (Sigma‐Aldrich, USA) in a ratio of 100:1. The reaction was performed at room temperature, under constant stirring, and pH was maintained in a range of 6–8 using sodium hydroxide (NaOH, 5 m) (AkzoNobel, USA) solution. After the reaction, synthetized PLMA were purified by dialysis with Float‐A‐Lyzer G2 Dialysis Device 3.5–5 kDa (Spectrum, USA) against deionized water for about 24 h. The PLMA solution was sterilized with low protein retention 0.2 µm filter (Enzymatic S. A., Portugal), frozen with liquid nitrogen, lyophilized (LyoQuest Plus Eco, Telstar, Spain) and stored at 4 °C until further use.

##### Compressive Mechanical Testing

PLMA hydrogels at 10, 15, and 20% (w/v), PEGDA hydrogels at 10% (w/v), and Matrigel were mechanically characterized by compression testing using the Instron 3340 Series Universal Testing System (Instron, USA) equipped with a 50 N load cell. The assays were performed on freshly prepared hydrogels with a cylindrical form (6 mm of diameter and 2.5 mm of height), at room temperature, except for Matrigel with which the assays were performed at 37 °C in order to maintain protein polymerization. The Young's modulus was defined as the slope of the linear region (0–5% of strain) of the strain–stress curve.

##### Cell Culture

hBM‐MSCs (ATCC, USA) and MG‐63 cell line (ECACC, Sigma‐Aldrich, USA) were cultured in minimum essential medium alpha (α‐MEM) (Thermo Fisher Scientific, USA) supplemented with sodium bicarbonate (2.2 g L^−1^, Sigma‐Aldrich, USA), 10% heat‐inactivated fetal bovine serum (FBS) (Thermo Fisher Scientific, USA), and 1% antibiotic/antimycotic (Thermo Fisher Scientific, USA). SaOS‐2 cell line (ECACC, Sigma‐Aldrich, USA) was cultured in Dulbecco's modified Eagle's medium (DMEM) low glucose (Sigma‐Aldrich, USA) supplemented with sodium bicarbonate (3.7 g L^−1^), 10% heat‐inactivated FBS, and 1% antibiotic/antimycotic. A549 cell line (ATCC, USA) was cultured with Nutrient Mixture F‐12 Ham (Sigma‐Aldrich, USA) supplemented with sodium bicarbonate (2.5 g L^−1^), 10% heat‐inactivated FBS, and 1% antibiotic/antimycotic. All cells were cultured in T‐flasks, maintained under 5% CO_2_ atmosphere at 37 °C (standard culture conditions) and passaged at about 80% confluence. The medium was replaced every 2 to 3 d.

##### Generation of Multicellular Spheroids

Each of the four cells types (hBM‐MSC, MG‐63, SaOS‐2, and A549) was detached with 0.25% trypsin/EDTA (Gibco, Thermo Fisher Scientific, USA) and resuspended in its culture medium. A density of 12 000 cells in culture medium (150 µL) was seeded onto 96‐well round‐bottom ultralow attachment plates (Corning, Thermo Fisher Scientific, USA) in order to generate spheroids. In the case of SaOS‐2 and A549 cell lines, the culture mediums were supplemented with 0.5% (w/v) of Methocel A4M (Sigma‐Aldrich, USA). Seeded cells were centrifuged at 500 × *g* for 10 min and incubated for 72 h at standard culture conditions. Generated spheroids were imaged by optical contrast light microscopy (Primostar, Carl Zeiss, Germany) using ZEN Imaging software.

##### 3D Invasion Assay

After 72 h of culture, generated spheroids were embedded into different hydrogel solutions: 10, 15, and 20% (w/v) PLMA100, 10% (w/v) PEGDA (*M*_n_ = 10 000 g mol^−1^, Sigma‐Aldrich, USA), and Matrigel Matrix (Corning, Thermo Fisher Scientific, USA). Embedding and culture of spheroids were performed into µ‐Slide Angiogenesis (ibidi, Germany) with one spheroid per well. PLMA hydrogel precursor solutions of 10, 15, and 20% (w/v) were prepared dissolving lyophilized PLMA in a sterilized solution of 0.5% (w/v) 2‐hydroxy‐4′‐(2‐hydroxyethoxy)‐2‐methylpropiophenone (Sigma‐Aldrich, USA), also known as Irgacure, in phosphate buffered saline (PBS) (Sigma‐Aldrich, USA). PEGDA hydrogel solution of 10% (w/v) was prepared following the same procedure and then sterilized with 0.2 µm filter. Matrigel Matrix solution (8–12 mg mL^−1^) was used as received from the provider. For PLMA and PEGDA‐based 3D invasion assays, a first layer of hydrogel solution (5 µL) was made by photopolymerization using ultraviolet (UV) irradiation (0.095 W cm^−2^) and a collimator during 15s, performing a semi‐crosslinking. The spheroid was deposited above, as much as possible in the center of the well, and a second layer was formed at the same conditions during 60 s for complete crosslinking. For Matrigel‐based 3D invasion assay, two layers were also performed, incubating the slides during 5 and 30 min at 37 °C for first and second layer polymerization, respectively. Thereafter, the appropriate culture medium (depending on cell type) was added to each well. The invasion systems were incubated for 14 d and the culture medium was replaced every 2 to 3 d.

a) *Cell viability analysis*: After 24 h and 14 d of spheroids embedding, a Live/Dead cell assay was performed for viability assessment. The hydrogels were incubated in a solution of 1:100 of Calcein AM solution in DMSO (4 × 10^−3^ m, Life Technologies, Thermo Fisher Scientific, USA) and 1:200 of propidium iodide (PI) (Thermo Fisher Scientific, USA) in PBS at standard culture conditions (5% CO_2_ at 37 °C) for 2 h. After washing with PBS, the 3D invasion models were observed under a fluorescence microscope (Fluorescence Microscope Zeiss, Axio Imager 2, Carl Zeiss, Germany).
b) *Cell morphology analysis*: Cell morphology assessment of invasive models was performed at 14 d of culture using a DAPI/phalloidin staining. At the aforementioned time‐point, hydrogels were washed with PBS and fixed in a 4% formaldehyde (Sigma‐Aldrich, USA) and 1% glutaraldehyde (Sigma‐Aldrich, USA) solution in PBS during at least 2 h. Before staining, samples were permeabilized with 0.5% Triton X‐100 for 30 min and blocked with 5% FBS in PBS for 1 h at room temperature. The hydrogels were firstly incubated in a phalloidin solution (Flash Phalloidin Red 594, Biolegend, USA) diluted 1:8 in PBS at room temperature for 90 min. After washing with PBS, a DAPI (5 µg mL^−1^, Thermo Fisher Scientific, USA) solution diluted 1:200 in PBS was prepared and used to incubate the hydrogels during 30 min at room temperature. After several PBS washes, the hydrogels were observed under a confocal fluorescence microscope (Zeiss LSM 510 META confocal laser scanning microscope, Carl Zeiss, Germany).
c) *Cell proliferation quantification*: Cell proliferation was assessed by ATP quantification using the CellTiter‐Glo 3D Cell Viability Assay (Promega, Madison, USA). First, all samples were washed with PBS, frozen at 80 °C in distilled water and thawed at 37 °C. PLMA hydrogels were then incubated with 1% trypsin/EDTA in distilled water (Gibco, Thermo Fisher Scientific, USA) for 1 h at 37 °C in order to expose the spheroid and invading cells. PEGDA hydrogels were mechanically dissociated in an ultrasound bath for about 10 min. PEGDA hydrogels and Matrigel were also incubated in 1% trypsin/EDTA in distilled water. Afterward, CellTiter‐Glo assay was performed in accordance with the manufacturer instructions. Briefly, CellTiter‐Glo reagent was added at a 1:1 ratio, the samples were vigorously mixed for 5 min and then incubated for 25 min at RT. Luminescence was measured in 96‐well flat‐bottom opaque white plates using Synergy HTX microplate reader (BioTek Instruments, Winooski, USA).

##### Quantification of Spheroid Size and Invasion Length

After spheroids embedding, cell spheroid invasion was monitored and imaged using an inverted optical contrast light microscope (Primostar, Carl Zeiss, Germany) with ZEN Imaging software. Image acquisition was performed every day until the third day and then at 7, 10, and 14 d of culture. The day 0 correspond to the pictures taken 1 h after spheroid embedding. Raw images were processed using the Image Processing tool of ZEN Image software. Quantification of spheroid area was performed using SpheroidSizer, a MATLAB‐based and open‐source high‐throughput image analysis software that applies an adapted active contour algorithm suitable to accurately measure spheroid size.^89^ The robustness of the algorithm allows an automatic or manual delimitation of the spheroid area, excluding the invasive branches. Quantification of spheroid invasion length was performed using ImageJ software. The spheroid center was estimated and the length from that point to the longer branch of each sample—invasion radius—was measured. The invasion length was normalized for each time‐point to the spheroid radius measured at day 0 by using Equation (1)

(1) $$\text{Invasion}\;\;\text{length}=\text{Invasion}\;\;\text{radius}-\text{Spheroid}\;\;{\text{radius}}_{\text{Day}0}$$

where Invasion radius is the length from the center of the spheroid to the end of the longer branch and the Spheroid radius _Day 0_ is the obtained value for the radius from the spheroid area measured at day 0, considering the spheroid delimitation a circumference. In both software, the sizes were converted from the acquired pixels (px), taking into account the objective amplification used, and expressed in micrometers (µm). Samples displaying post‐embedding abnormalities related with their location inside the hydrogel or lack of invasion at the end of the experiment were excluded.

##### Quantification of Protein and Growth Factors Release

The protein release assays were performed in PLMA hydrogels at 10, 15, and 20% (w/v) without encapsulated spheroids. The samples (*n* = 6) were placed into falcons with PBS (5 mL, Thermo Fischer Scientific, USA) and incubated with constant agitation (60 rpm) in a water bath at 37 °C. Over 14 d, an aliquot (1 mL) was taken at each time‐point and fresh PBS (1 mL) was added. The collected aliquots were stored at −20 °C. For total protein quantification, Micro BCA Protein Assay Kit (Thermo Fisher Scientific, USA) was used. ELISA assays were performed to quantify the release of vascular endothelial growth factor (VEGF Human ELISA Kit, Invitrogen, ThermoFisher Scientific, USA), transforming growth factor β1 (TGF‐β1 Human ELISA Kit, Invitrogen, ThermoFisher Scientific, USA), and epidermal growth factor (Human EGF Quantikine ELISA Kit, R&D systems, Minneapolis, USA).

##### Statistical Analysis

All data were statistically analyzed using GraphPad Prism 7 Software and are expressed as mean ± standard deviation (SD) or mean ± standard error of the mean (SEM) of at least three independent experiments. The distribution of the initial spheroid sizes was analyzed with the D'Agostino–Pearson normality test. For invasion speed data, statistical significance between the different groups was identified using two‐way ANOVA analysis of variance combined with Tukey's multiple comparisons test, and the differences were considered significant when *p* < 0.05. Statistical significance for Young's modulus and ATP quantification data was evaluated by one‐way ANOVA analysis using Tukey's multiple comparisons test.

## Conflict of Interest

The authors declare no conflict of interest.

## Supporting information

Supporting Information
