# Supplementary material for: Human Platelet Lysates‐Based Hydrogels: A Novel Personalized 3D Platform for Spheroid Invasion Assessment
Source: Adv Sci (Weinh). 2020 Feb 11;7(7):1902398. doi: 10.1002/advs.201902398 (PMC7141025; doi:10.1002/advs.201902398)
Supplement: Supplementary file 1 — Supporting Information [file ADVS-7-1902398-s001.pdf]

Copyright WILEY-VCH Verlag GmbH & Co. KGaA, 69469 Weinheim, Germany, 2020.

ADVANCED SCIENCE
Open Access

Supporting Information

for *Adv. Sci.,* DOI: 10.1002/advs.201902398

Human Platelet Lysates-Based Hydrogels: A Novel Personalized 3D Platform for Spheroid Invasion Assessment

Cátia F. Monteiro, Sara C. Santos, Catarina A. Custódio, and João F. Mano**

Copyright WILEY-VCH Verlag GmbH & Co. KGaA, 69469 Weinheim, Germany, 2020.

Supporting Information

Human Platelet Lysates-Based Hydrogels – A Novel Personalized 3D Platform for Spheroid Invasion Assessment

Cátia F. Monteiro, Sara C. Santos, Catarina A. Custódio, João F. Mano**

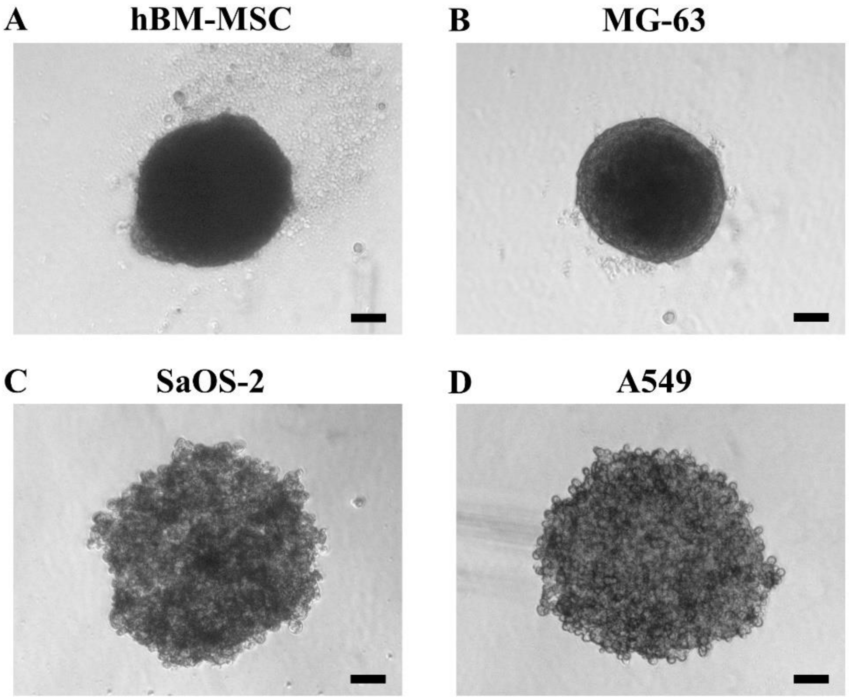

Figure S1. Optical microscopy images of different cell spheroid types. Spheroids of **(A)** hBM-MSC, **(B)** MG-63, **(C)** SaOS-2 and **(D)** A549 generated during 3 days, from an initial number of 12 000 cells/spheroid. Scale bar: 100 µm

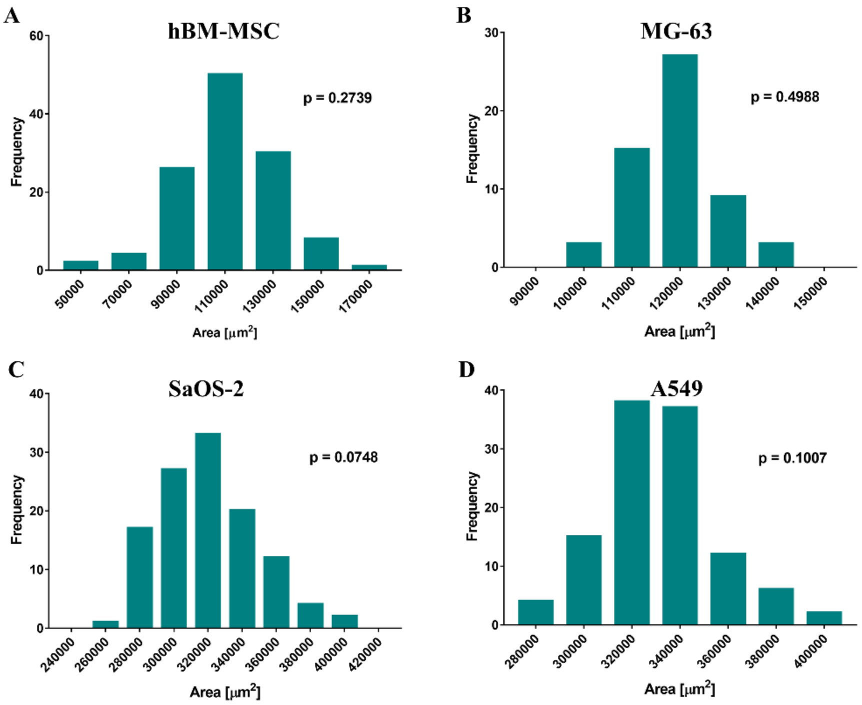

Figure S2. Distribution of the initial spheroid sizes. Spheroids generated in round-bottom ultra-low attachment plates from an initial number of 12 000 cells of **(A)** hBM-MSC, **(B)**, MG-63, **(C)** SaOS-2 and **(D)** A549, were measured and their initial sizes were analyzed with the D-Agostino-Pearson normality test. p value was obtained through the normality test and a normal distribution is considered when $p>0.05$.

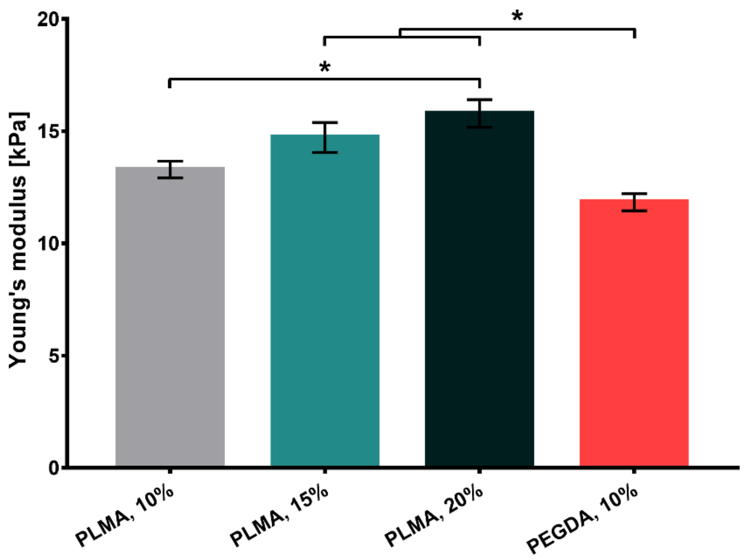

Figure S3. Young's modulus for PLMA hydrogels at 10, 15 and 20% (w/v) , PEGDA hydrogels at 10% (w/v) and Matrigel®. # means significant differences with the groups on the right. Statistically significant differences are indicated: *p<0.1. Data is presented as mean ± SEM (n≥3).

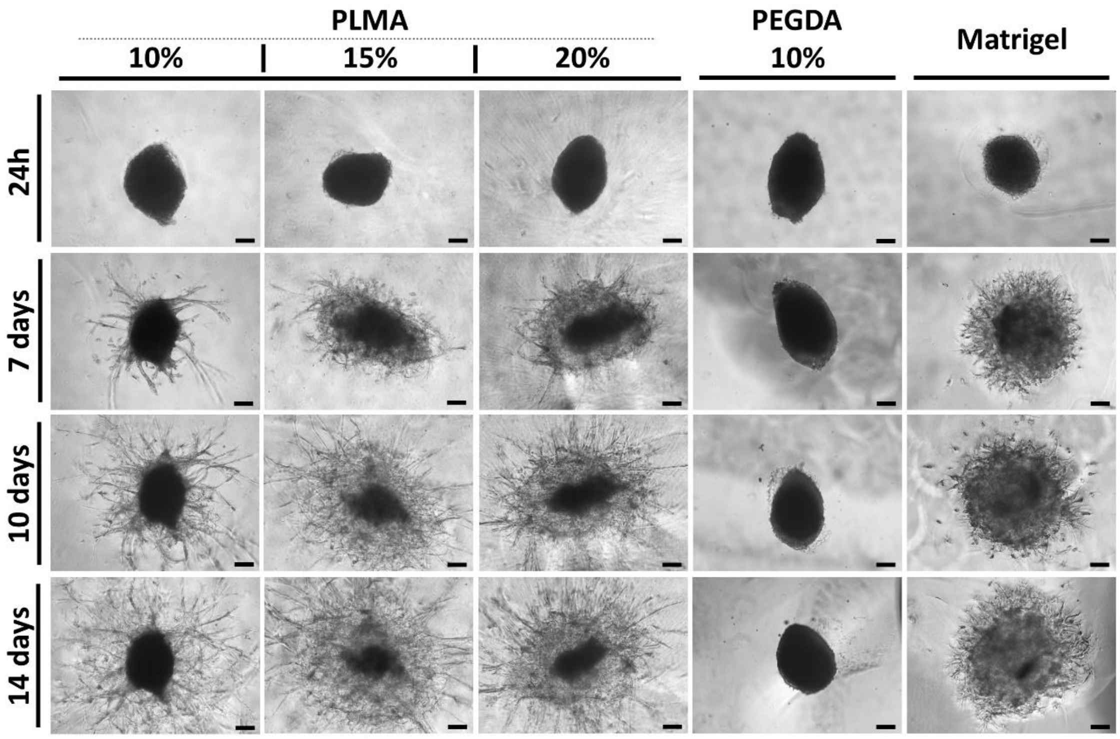

Figure S4. hBM-MSC spheroids encapsulated into the three different biomaterials (PLMA at 10, 15 and 20% (w/v), PEGDA 10% (w/v) and Matrigel®). Morphological and invasiveness analysis of hBM-MSC spheroids over time by optical contrast microscopy. All spheroids were generated with an initial number of 12 000 cells per spheroid. Scale bar: 100 µm.

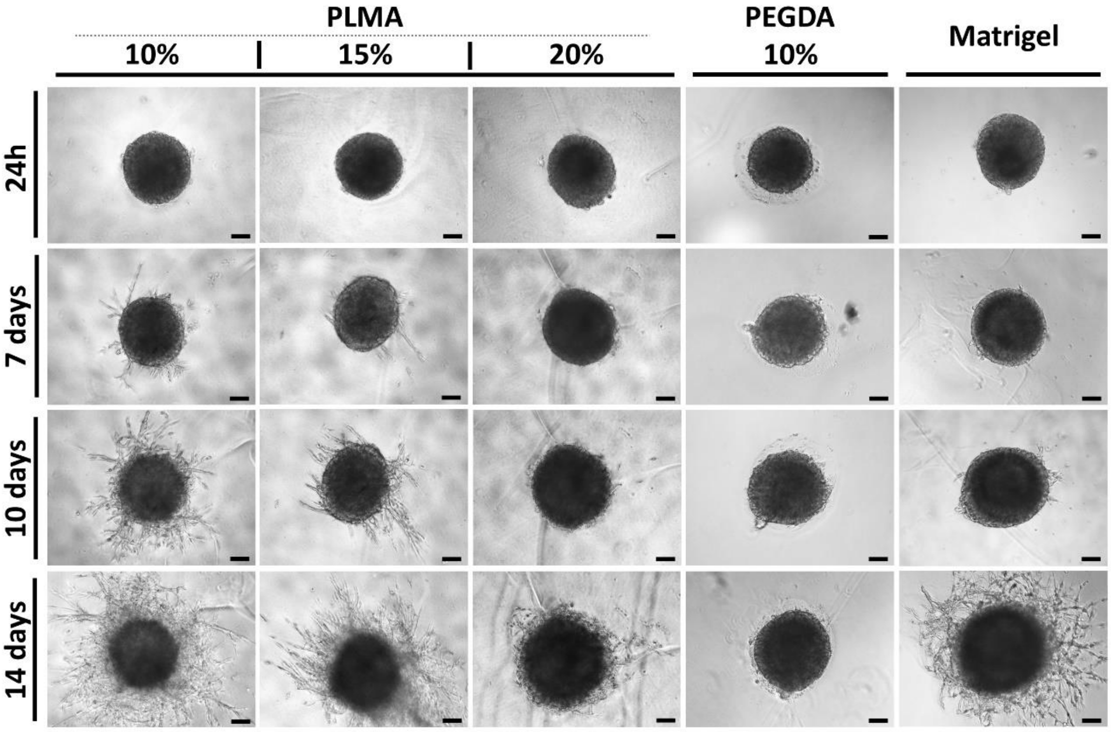

Figure S5. MG-63 spheroids encapsulated into the three different biomaterials (PLMA at 10, 15 and 20% (w/v), PEGDA 10% (w/v) and Matrigel®). Morphological and invasiveness analysis of MG-63 spheroids over time by optical contrast microscopy. All spheroids were generated with an initial number of 12 000 cells per spheroid. Scale bar: 100 µm.

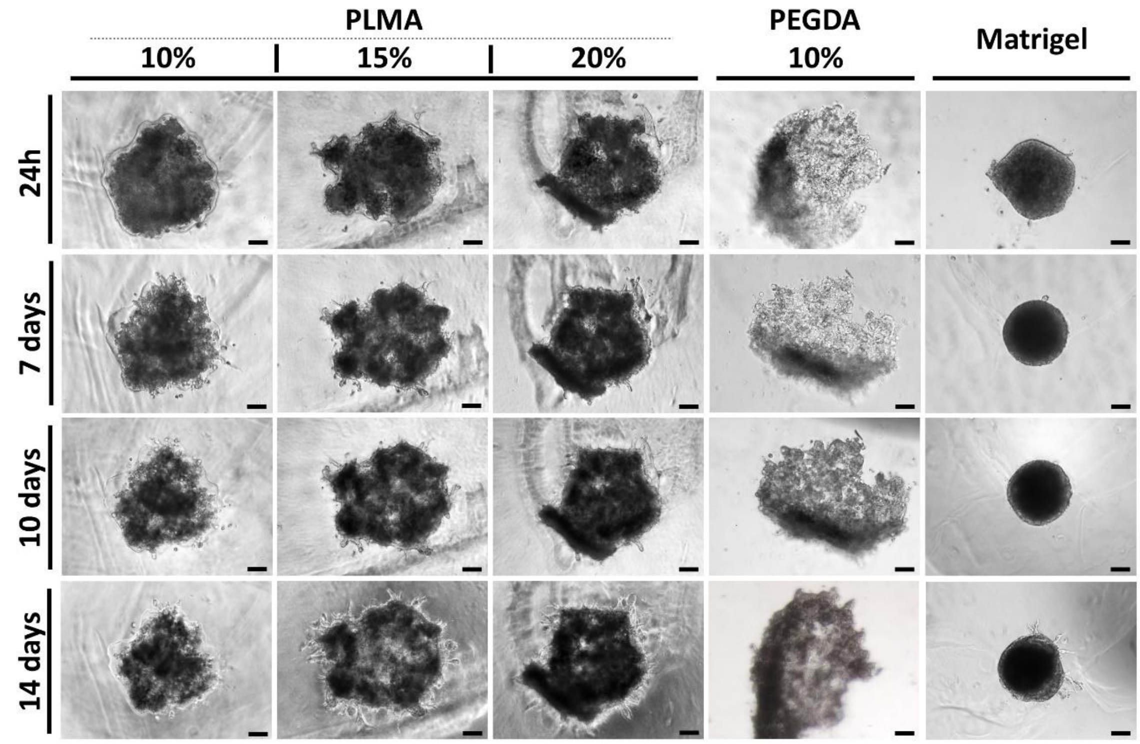

Figure S6. SaOS-2 spheroids encapsulated into the three different biomaterials (PLMA at 10, 15 and 20% (w/v), PEGDA 10% (w/v) and Matrigel®). Morphological and invasiveness analysis of SaOS-2 spheroids over time by optical contrast microscopy. All spheroids were generated with an initial number of 12 000 cells per spheroid. Scale bar: 100 μm.

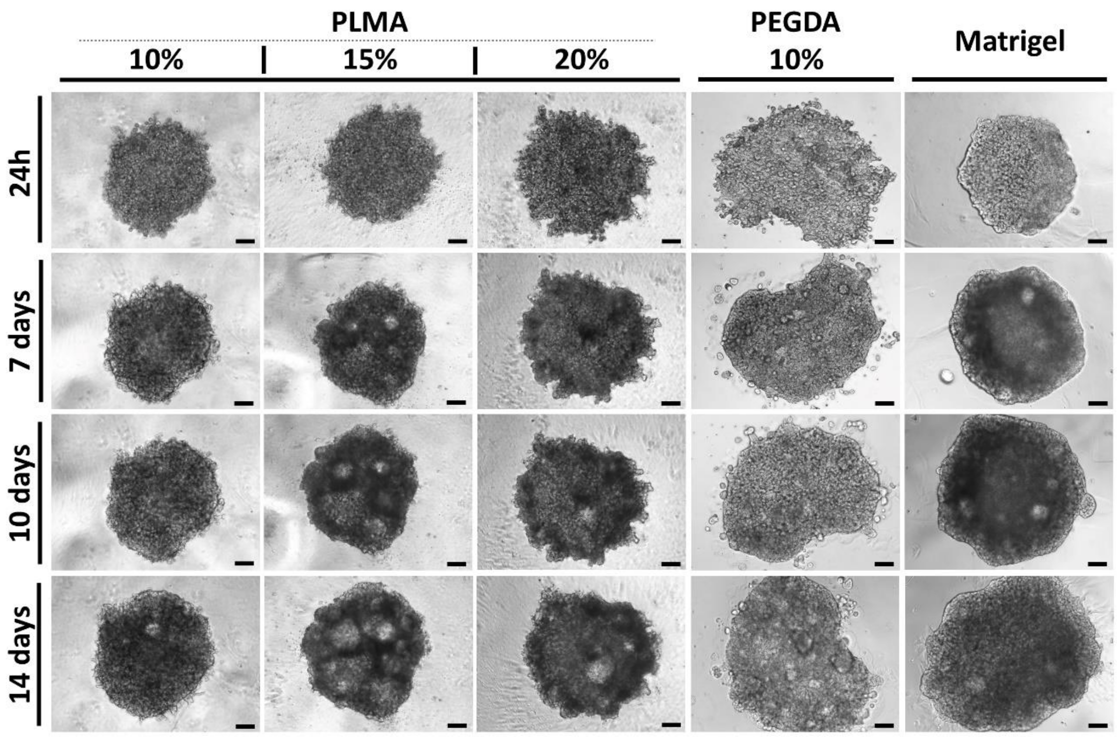

Figure S7. A549 spheroids encapsulated into the three different biomaterials (PLMA at 10, 15 and 20% (w/v), PEGDA 10% (w/v) and Matrigel®). Morphological and invasiveness analysis of A549 spheroids over time by optical contrast microscopy. All spheroids were generated with an initial number of 12 000 cells per spheroid. Scale bar: 100 μm.

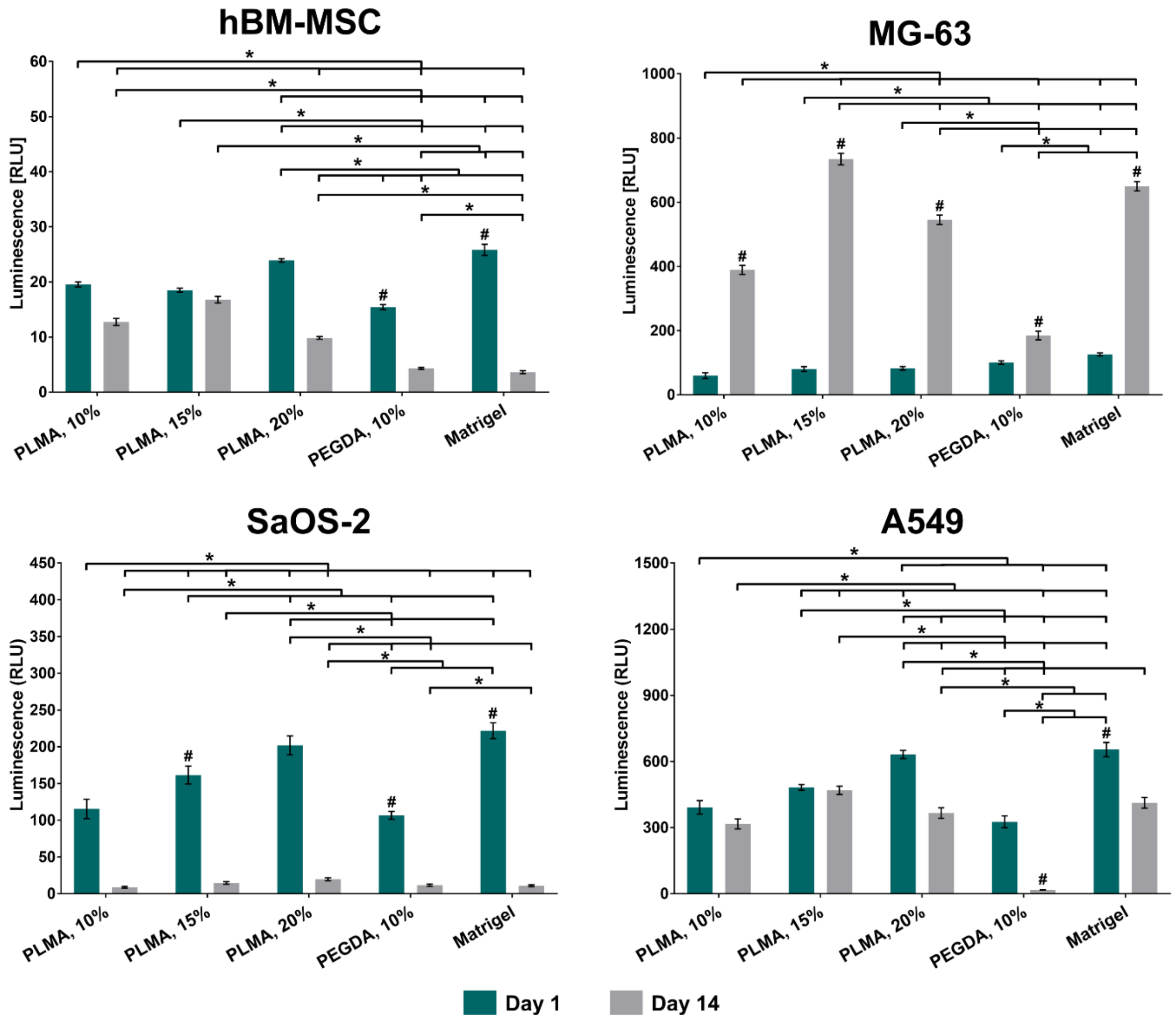

Figure S8. ATP quantification of each spheroid type into the different biomaterials and stiffness conditions, at 24h and 14 days of culture. Results are expressed in relative luminescence units (RLU), and statistically significant differences are indicated: *p<0.1. Data is presented as mean ± SEM (n≥3).

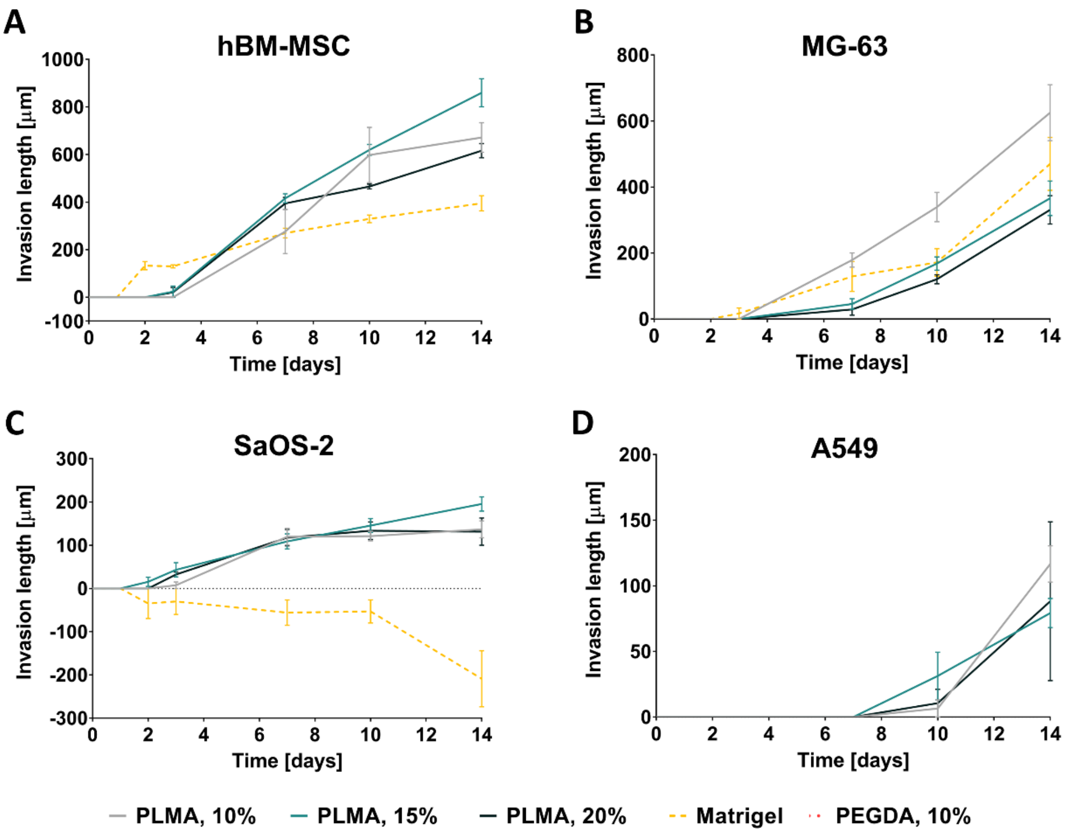

Figure S9. Invasion length progression of (A) hBM-MSC, (B) MG-63, (C) SaOS-2 and (D) A549 spheroids over the 14 days of culture. The invasion length was determined considering the invasion radius from spheroid center to the longer branch of invasion and normalizing that value to the spheroid radius measured at day 0. All spheroids were generated with an initial number of 12 000 cells per spheroid. Data is presented as mean ± SD (n≥3).

Table S1. Goodness of fit (R^2) of invasion kinetics for each condition. Data results from a linear regression analysis of the invasion length up to 14 days of culture and R^2 is presented as mean ± SD (n≥3). Conditions that presented R^2<0.80 are in gray.

	PLMA			PEGDA	Matrigel
	**10%**	**15%**	**20%**	**10%**	
**hBM-MSC**	0.935 ± 0.007	0.967 ± 0.002	0.934 ± 0.039	n.d.	0.901 ± 0.054
**MG-63**	0.940 ± 0.011	0.862 ± 0.058	0.906 ± 0.039	n.d.	0.852 ± 0.076
**SaOS-2**	0.835 ± 0.127	0.938 ± 0.027	0.818 ± 0.131	n.d.	0.691 ± 0.115
**A549**	0.571 ± 0.057	0.680 ± 0.138	0.678 ± 0.136	n.d.	n.d.
